# Physiological Adaptations and Thermoregulatory Indicators of Heat Stress in Bovine Production Systems

**DOI:** 10.3390/ani16193003

**Published:** 2026-09-24

**Authors:** Ramiro F. Díaz, Samantha Isabel Román Larco, Kriss Aguilar, Andrés Caicedo

**Affiliations:** 1 Escuela de Medicina Veterinaria, Universidad San Francisco de Quito USFQ, Quito 170901, Ecuador; sromanl@estud.usfq.edu.ec (S.I.R.L.); ksaguilar@estud.usfq.edu.ec (K.A.); 2 Instituto de Investigaciones en Biomedicina iBioMed, Universidad San Francisco de Quito USFQ, Quito 170901, Ecuador; acaicedo@usfq.edu.ec; 3 Mito-Act Research Consortium, Quito 17090, Ecuador; 4 Escuela de Medicina, Colegio de Ciencias de la Salud, Universidad San Francisco de Quito USFQ, Quito 170901, Ecuador

**Keywords:** thermoregulation, reproduction, heat stress, temperature–humidity index

## Abstract

Heat stress is a major challenge for cattle production systems because it negatively affects animal welfare, health, productivity, and reproductive performance. This communication reviews the main physiological adaptations and thermoregulatory indicators associated with heat stress in cattle and illustrates their practical application under tropical conditions. Environmental and physiological measurements were evaluated in fifty adult *Bos indicus* cows in Tabasco, Mexico. Ambient temperature was recorded using black globe and dry-bulb thermometers under different environmental conditions, while rectal temperature was used as an indicator of internal heat load. Black globe temperatures measured under direct solar radiation were significantly higher than those recorded under shaded conditions (*p* < 0.05), particularly between 10:00 and 14:00 h. Rectal temperature increased from 38.73 ± 0.73 °C during the morning to 41.24 ± 2.00 °C during periods of maximum environmental heat load (*p* = 0.001). These findings demonstrate the close relationship between environmental heat load and thermoregulatory responses in cattle. Monitoring indicators such as black globe temperature and rectal temperature may provide practical tools for the early detection of heat stress and support management strategies aimed at improving animal welfare and production efficiency under tropical conditions.

## Simple Summary

Heat stress is one of the major challenges affecting cattle production worldwide, particularly in tropical and subtropical regions. When environmental temperatures exceed the animal’s capacity to dissipate heat, physiological mechanisms are activated to maintain body temperature, often compromising productivity, reproduction, health, and welfare. Identifying reliable thermoregulatory indicators is therefore essential for early detection and management of heat stress. This communication reviews the principal physiological responses and biomarkers associated with thermal stress in cattle and illustrates their practical application through a field study conducted in *Bos indicus* cows under tropical conditions in Tabasco, Mexico. Environmental temperatures measured using black globe thermometers were significantly higher under direct solar radiation than under shaded conditions, and increases in ambient heat load were associated with elevated rectal temperatures. These findings highlight the importance of combining environmental and physiological indicators to improve heat stress monitoring and support management strategies aimed at enhancing animal welfare and production efficiency in warm climates.

## 1. Introduction

Heat stress has become one of the greatest environmental challenges affecting cattle production worldwide. The increasing frequency, intensity, and duration of heat waves associated with climate change are compromising animal welfare, health, reproductive efficiency, and productive performance in both dairy and beef production systems [1,2]. These effects are particularly relevant in tropical and subtropical regions, where high ambient temperatures frequently coincide with elevated humidity and solar radiation, increasing the thermal load imposed on animals.

Heat stress occurs when the total heat load absorbed by an animal exceeds its capacity to dissipate heat and maintain thermal homeostasis [1]. Under these conditions, cattle activate a series of behavioral, physiological, endocrine, and metabolic mechanisms aimed at maintaining core body temperature within narrow limits. These responses include increased respiratory rate, peripheral vasodilation, reduced feed intake, alterations in endocrine function, and changes in energy metabolism [3]. Although these adaptive mechanisms are essential for survival, prolonged or severe heat stress may impair immune function, reproductive performance, productivity, and ultimately animal welfare [4].

The magnitude of the heat stress response is not uniform among mammals. Breed, age, physiological status, body condition, coat characteristics, production level, and previous acclimation influence individual susceptibility to thermal stress [5]. *Bos indicus* cattle, for example, generally exhibit greater heat tolerance than *Bos taurus* breeds because of morphological, physiological, and behavioral adaptations that improve heat dissipation [3,5]. Consequently, evaluating heat stress requires consideration of both environmental conditions and animal-based physiological responses.

Reliable thermoregulatory indicators are therefore essential for early detection and monitoring of heat stress under field conditions. Environmental indices and physiological biomarkers provide valuable information regarding the interaction between climatic conditions and the animal’s thermoregulatory capacity, allowing producers and veterinarians to implement timely management strategies aimed at reducing thermal stress and improving welfare and productive efficiency [4,6].

Although numerous studies have described the physiological consequences of heat stress in cattle, integrating these concepts with practical field indicators remains essential for improving on-farm monitoring and decision-making. In this communication, reviews the principal physiological adaptations and thermoregulatory indicators associated with heat stress in cattle and illustrate their practical application using field observations obtained from *Bos indicus* cows raised under tropical environmental conditions in Tabasco, Mexico.

Therefore, the objective of this communication is to describe the principal physiological responses associated with heat stress, discuss the most useful thermoregulatory indicators currently available for cattle, and demonstrate their practical application through field observations obtained under tropical production conditions.

## 2. Key Physiological Responses to Heat

Heat stress develops when the total environmental and metabolic heat load exceeds the animal’s capacity to dissipate heat while maintaining a stable core body temperature. This condition generally occurs when ambient temperature, humidity, solar radiation, and wind speed combined to produce a thermal load that surpasses the animal’s upper critical temperature, forcing activation of compensatory thermoregulatory mechanisms [7]. The severity of heat stress depends not only on environmental conditions but also on the duration of exposure, animal characteristics, and the effectiveness of heat dissipation mechanisms.

Physiological responses to heat stress occur in a hierarchical manner and may be classified into autonomic, metabolic, endocrine, and cellular responses. Their primary objective is to maintain thermal homeostasis while minimizing tissue damage. However, when heat exposure is prolonged or particularly intense, these adaptive responses become progressively less effective, leading to impaired physiological function and reduced productive performance.

The thermal balance equation indicates that metabolic heat production together with environmental heat gain must be balanced by heat dissipation through conduction, convection, radiation, and evaporation [7]. Under moderate environmental conditions, sensible heat loss predominates. However, as ambient temperature approaches or exceeds body temperature, evaporative cooling through sweating and increased respiratory evaporation becomes the principal mechanism of heat dissipation [7,8].

One of the earliest physiological responses is peripheral vasodilation, which increases blood flow to the skin and facilitates heat exchange with the environment [8,9]. Simultaneously, respiratory rate increases, allowing greater evaporative heat loss through the respiratory tract. As heat load intensifies, cattle progressively reduce voluntary feed intake, thereby decreasing metabolic heat production associated with digestion and nutrient metabolism [9].

These physiological adjustments are accompanied by endocrine adaptations. Plasma concentrations of thyroid hormones, particularly triiodothyronine (T3) and thyroxine (T4), decrease during prolonged heat exposure, reflecting a reduction in basal metabolic rate and endogenous heat production [10]. These hormones are secreted by the thyroid gland under regulation of the hypothalamic-pituitary-thyroid axis and play a central role in controlling metabolic activity. Their reduction represents an adaptive mechanism that limits internal heat generation during periods of thermal stress [10,11].

In parallel, activation of the hypothalamic–pituitary–adrenal (HPA) axis increases circulating glucocorticoids, mainly cortisol, which redistribute energy resources toward essential physiological processes required for survival [10]. Although cortisol is a general stress hormone, sustained activation of the HPA axis during heat stress contributes to metabolic adjustments, immunomodulation, and maintenance of homeostasis under elevated thermal load [12]. Concurrently, heat shock proteins, particularly HSP70, are synthesized in response to elevated cellular temperatures and function as molecular chaperones that stabilize proteins, prevent protein denaturation, and facilitate cellular repair [13].

At the cellular level, prolonged or severe heat stress, defined as sustained exposure to environmental conditions that exceed the animal’s capacity to maintain thermal homeostasis despite activation of thermoregulatory mechanisms, induces oxidative stress, mitochondrial dysfunction, protein misfolding, membrane instability, and apoptosis when cellular repair mechanisms become insufficient [14]. Antioxidant enzymes, including superoxide dismutase and glutathione peroxidase, are upregulated to limit oxidative damage, although their protective capacity may be exceeded during prolonged exposure [15].

Heat stress also affects reproductive physiology. Elevated body temperature compromises follicular development, oocyte competence, embryo survival, spermatogenesis, and placental function through endocrine alterations and direct cellular damage [10]. Consequently, reproductive efficiency may remain impaired even after environmental conditions have returned to normal.

Repeated exposure to elevated temperatures induces acclimation, a reversible physiological adjustment that develops over days or weeks within the lifetime of an individual animal. Acclimation improves heat dissipation by increasing sweating efficiency, optimizing cardiovascular responses, and modifying endocrine regulation [16]. In contrast, adaptation refers to heritable genetic changes that occur over many generations through natural or artificial selection. *Bos indicus* cattle are an example of evolutionary adaptation to hot environments, exhibiting morphological and physiological characteristics such as lighter coats, greater skin surface area, lower subcutaneous fat deposition, and more efficient sweating mechanisms than *Bos taurus* cattle [17]. Nevertheless, intensive selection for high productive performance may reduce thermotolerance, creating a biological trade-off between productivity and resilience to heat stress.

## 3. Thermoregulatory Markers (Indicators) of Heat Stress

To understand, monitor or select for heat resilience, one must identify reliable thermoregulatory indicators. Such markers can be core body temperature measures and surface temperature indices respiratory variables, blood or endocrine biomarkers, and behavioral/physiological composites.

### 3.1. Core Body Temperature Metrics and Surface and Skin Temperature Indices

Rectal temperature is perhaps the most classic index of core temperature, but it is invasive, subject to handling artefacts, and slow to reflect rapid changes [18]. Alternatives include vaginal temperature, ruminal (reticulo-ruminal) temperature probes, tympanic (ear) sensors, and ingestible telemetric boluses. These approaches differ in response time, invasiveness, cost, and practicality [19]. Telemetric boluses can provide continuous high-resolution internal temperature data, thereby capturing fluctuations and cumulative heat load better than point measurements.

Surface temperature can be assessed non-invasively using infrared thermography (IRT), which records the infrared radiation emitted by the skin to generate two-dimensional temperature maps of specific body regions [20]. Unlike core body temperature measurements (e.g., rectal, vaginal, or ruminal temperature), which directly reflect the animal’s internal thermal status, IRT evaluates peripheral skin temperature and therefore provides indirect information about thermoregulatory responses. Changes in surface temperature primarily reflect alterations in peripheral blood flow, cutaneous vasodilation, and heat dissipation rather than core body temperature itself [20].

In cattle, infrared thermography of the ocular region, muzzle, udder, and flanks have shown significant associations with rectal temperature, respiratory rate, and other physiological indicators of heat stress, making it a valuable tool for early, non-invasive assessment of thermal status [21]. However, because surface temperature is strongly influenced by environmental conditions such as solar radiation, wind speed, ambient temperature, coat characteristics, and hair thickness, infrared thermographic measurements require careful calibration and standardized measurement protocols to ensure reliable interpretation [22]. Consequently, infrared thermography should be considered complementary to core body temperature measurements rather than a direct replacement, particularly when evaluating heat stress under field conditions.

A research study was conducted during the summer months (June) in the state of Tabasco, Mexico, using rectal and infrared measurements to determine the relationship between ambient temperature and body temperature in *Bos indicus* cows, as described below.

#### 3.1.1. Thermoregulatory Responses of Bos indicus Cows Under Tropical Conditions

##### Materials and Methods

The field study was conducted during the summer (June) at the Experimental Center of the Colegio de Postgraduados (COLPOS), Campus Tabasco, Mexico, under tropical environmental conditions. Fifty clinically healthy adult Bos indicus cows were included in the study.

Environmental temperature was monitored over a 24-h period using a black globe thermometer placed under three environmental conditions: direct solar radiation (TBGS), tree shade (TBGT), and roofed shade (TBGR). Simultaneously, ambient temperature was measured with a dry-bulb thermometer under direct solar radiation (TDBS) and tree shade (TDBSh).

Core body temperature was determined using a veterinary rectal thermometer (Sinteg^®^, Mexico City, Mexico). A total of 31 rectal temperature measurements were obtained, including 14 measurements between 09:00 and 11:00 h and 17 measurements between 12:00 and 14:00 h, corresponding to the period of greatest environmental heat load.

Body surface temperature was recorded simultaneously using a Fluke 64 MAX infrared thermometer (Fluke Corporation, Everett, WA, USA), a non-contact infrared thermometer with a measurement range of −30 to 600 °C, an accuracy of ±1.0 °C or ±1% of the reading (whichever is greater), a 20:1 distance-to-spot ratio, and laser targeting to improve measurement precision. Infrared temperature was obtained immediately after rectal temperature measurements under the same environmental conditions. The technical specifications of the materials used are listed in Table 1.

Environmental temperatures and body temperature measurements were analyzed using repeated-measures analysis of variance (ANOVA). Mean values are presented as mean ± standard deviation (SD), and differences among environmental conditions or sampling periods were considered statistically significant at *p* < 0.05. Statistical analyses were performed using IBM SPSS Statistics version 22.0 (IBM Corp., Armonk, NY, USA).

##### Results

Black globe temperature differed significantly among environmental conditions only during the period of maximum environmental heat load (10:30–14:00 h). During this interval, temperatures recorded under direct solar radiation (TBGS) were significantly higher (42.12 ± SD °C; *p* < 0.05) than those measured under tree shade (33.62 ± SD °C) and roofed shade (33.25 ± SD °C), whereas no significant differences were detected between the two shaded conditions (Table 2). Outside this period, no significant differences (*p* > 0.05) were observed among the three environmental conditions.

The temperatures detected by the black globe were not affected by wind and humidity (the globe isolated the thermometer bulb from these environmental factors). In contrast, the dry bulb measurements were affected by humidity and wind. Thus, it was observed that the mean environmental temperatures recorded using the black globe thermometer under roofed shade (TBGR) did not differ significantly (*p* > 0.05) from those obtained using dry-bulb thermometers under tree shade (TDBSh) or direct solar radiation (TDBS) (Table 3). On the other hand, during the period of maximum environmental heat load (10:00–14:00 h), temperatures recorded using the black globe thermometer under direct solar radiation (TBGS) were significantly higher than those obtained with the dry-bulb thermometer (TDBS) (*p* < 0.05) (Table 4). No significant differences between the two measurement methods were observed during the remaining sampling period (*p* > 0.05). These results indicate that the black globe thermometer better reflects the combined effects of solar radiation and ambient conditions than the dry-bulb thermometer under direct sunlight.

Rectal temperatures varied depending on the time of day they were taken. In the morning (14 measurements between 9:00 and 11:00 a.m.), they averaged 38.73 °C ± 0.73. In contrast, in the afternoon (17 measurements between 13:00 and 14:00), they averaged 41.24 °C ± 2.0, which was significantly different (*p* = 0.001; Table 5). On the other hand, infrared temperatures were lower than rectal temperatures (*p* < 0.05) between 10:30 am and 11:50 am. It was during this time that rectal temperature reached its highest values of the day.

In this experiment, it was observed how body temperatures are consistent with ambient temperatures and, in turn, how ambient temperatures need to be influenced by wind and humidity to be more conducive to adaptation by animals living in tropical areas.

Environmental temperature significantly influenced the body temperature of adult Bos indicus cows, with higher rectal temperatures recorded during the hottest hours of the day. Black globe temperature measured under direct sunlight was strongly associated with rectal temperature, indicating that it is a useful indicator of the thermal load experienced by animals in tropical environments. Infrared thermometry underestimated body temperature compared with rectal measurements during periods of elevated heat load, suggesting that caution should be exercised when using this technique as a substitute for rectal thermometry.

The findings highlight the importance of effective thermoregulatory mechanisms in Bos indicus cattle and support the use of black globe temperature as a practical tool for assessing environmental conditions associated with heat stress risk.

### 3.2. Respiratory and Panting Variable

Respiratory rate (breaths per minute) is among the most frequently used animal-based physiological indicators of heat stress because it responds rapidly to increases in environmental heat load [23]. As ambient temperature rises, cattle increase respiratory frequency to enhance evaporative heat loss through the respiratory tract, making respiratory rate one of the earliest physiological responses to thermal challenges. Under thermoneutral conditions, respiratory rates generally range between 20 and 40 breaths per minute. During mild to moderate heat stress, respiratory rates commonly increase to approximately 60–80 breaths per minute, whereas animals experiencing severe heat stress may exceed 100 breaths per minute as evaporative cooling becomes the primary mechanism of heat dissipation [23,24]. Although these values may vary according to breed, production stage, acclimation, and environmental conditions, progressive increases in respiratory rate provide a practical indicator of increasing thermal load.

The panting score, a categorical assessment of respiratory effort, is also widely used in field conditions because it provides a rapid and practical estimate of the severity of heat stress [24]. However, panting is not specific to heat stress and may also occur in response to other stressors, including transportation, handling, pain, fear, or intense physical activity. Therefore, panting scores should always be interpreted together with environmental conditions and complementary physiological indicators, such as rectal temperature, body surface temperature, or temperature–humidity indices, to avoid misclassification of the animal’s thermal status.

Changes in tidal volume and the inspiratory-to-expiratory ratio may provide additional information regarding respiratory adaptation to heat stress. However, their assessment requires specialized instrumentation, such as pneumotachometers, limiting their routine application under commercial field conditions.

### 3.3. Haematological and Blood Biomarkers

Blood biomarkers provide valuable information regarding the systemic physiological response to heat stress beyond external thermoregulatory changes. Several biomarkers, including cortisol, glucose, non-esterified fatty acids, lactate, heat shock proteins (e.g., HSP70 and HSP90), thyroid hormones (T3 and T4), and oxidative stress markers such as malondialdehyde and antioxidant enzyme activities, have been proposed as useful indicators of the animal’s response to elevated thermal load. However, it is important to recognize that many of these biomarkers are not specific to heat stress. Hormonal, metabolic, and hematological alterations may also occur in response to transportation, handling, disease, pain, nutritional challenges, or other physiological stressors. Consequently, these variables should be interpreted as indicators of systemic stress rather than exclusive biomarkers of thermal stress [25]. Their greatest value lies in their combined interpretation with environmental measurements and animal-based physiological indicators such as rectal temperature, respiratory rate, or infrared thermography.

Electrolyte disturbances, including alterations in sodium, potassium, and bicarbonate concentrations, may reflect compensatory renal and respiratory mechanisms activated during prolonged heat exposure [26]. Likewise, leukocyte counts and other immune parameters may indicate immunosuppression associated with chronic heat stress, although similar changes may also occur under other stressful conditions [27].

Although blood biomarkers provide important information regarding the physiological consequences of heat stress, blood sampling is invasive, may itself induce an acute stress response, and only provides information at a single point in time. Therefore, these biomarkers are most informative when integrated with continuous environmental and physiological monitoring.

### 3.4. Composite Indices and Derived Indicators

Because no single physiological variable completely reflects the complexity of heat stress, composite environmental indices are commonly used to estimate the thermal challenge experienced by cattle. The Temperature–Humidity Index (THI) is the most widely applied environmental indicator because it combines ambient temperature and relative humidity into a single value that estimates the potential risk of heat stress [28].

One of the most frequently used equations for calculating THI in cattle is:

THI = (1.8 × T + 32) − [(0.55 − 0.0055RH)(1.8T − 26)]

THI provides a practical estimate of the environmental heat load and has been widely used to establish management thresholds in cattle production systems. Although threshold values may vary according to breed, production level, acclimation, and environmental conditions, dairy cattle generally begin to experience measurable physiological responses when THI exceeds 68, with increasing severity as THI rises (Table 6). More comprehensive environmental indices, such as the Heat Load Index (HLI) and the Black Globe Humidity Index (BGHI), additionally incorporate solar radiation, wind speed, or black globe temperature to better characterize the thermal environment under field conditions [28].

More recently, increasing attention has been directed toward integrated animal-based indices that combine environmental measurements with physiological variables, including respiratory rate, rectal temperature, body surface temperature, and infrared thermography, allowing earlier and more sensitive detection of heat stress than environmental measurements alone [29].

## 4. Monitoring Strategies and Practical Evaluation in Production Systems

Translating theoretical markers into practical, on-farm monitoring involves technical, economic, and logistical challenges. Optimized strategies are presented in this section.

Traditional monitoring relies on periodic manual measurements (rectal temperature, respiration counts, behavioral observation). While simple, this approach is labor-intensive, disruptive, and limited in temporal resolution. Advances in precision livestock farming allow automated, continuous monitoring of core or surface temperatures, respiration, or movement using sensors, wearable devices, and remote thermal cameras [30]. Such systems enable early warning and individual-targeted interventions. However, costs, data processing needs, sensor robustness, and farm infrastructure remain barriers.

Effective use of thermoregulatory indicators for monitoring heat stress requires establishing baseline physiological profiles according to breed, production stage, age, season, and local environmental conditions. These reference values allow the identification of deviations specifically associated with increasing thermal load rather than with normal physiological variation or other non-thermal stressors. For example, the relationship between respiration rate or core body temperature and the Temperature–Humidity Index (THI) has been used to distinguish heat-tolerant from heat-sensitive individuals [31]. Thresholds for intervention should therefore be adjusted according to local climate, breed characteristics, coat type, and housing conditions to improve the accuracy of heat stress detection. Without proper calibration, monitoring systems may generate false alarms or fail to identify animals experiencing thermal stress.

Continuous high-frequency monitoring enables the detection of transient increases in environmental heat load and physiological responses that may not be captured by periodic measurements. A combined monitoring strategy, integrating continuous measurements of core body temperature with periodic clinical evaluations, may provide the most reliable assessment of heat stress. Likewise, data smoothing, trend analysis, and anomaly detection algorithms are essential to distinguish genuine heat stress events from normal physiological fluctuations or responses to unrelated stressors.

Integrating animal-based physiological measurements with microclimatic variables, including ambient temperature, relative humidity, solar radiation, and wind speed, enables more accurate estimation of the effective thermal load experienced by cattle. Recent advances in predictive modelling and machine learning have improved the ability to forecast when animals are likely to reach critical heat stress thresholds and to automatically activate mitigation strategies, such as fans or sprinkler systems [32]. Such proactive management is more effective than responding only after clinical signs of heat stress become evident. Nevertheless, monitoring systems must be properly validated, routinely calibrated, and sufficiently robust for field conditions. In addition, user-friendly interfaces, automated alerts, and decision-support tools are essential for translating sensor data into practical management actions, while cost–benefit analyses remain necessary to ensure the economic feasibility of implementing precision heat stress monitoring systems.

## 5. Productive, Health and Welfare Implications of Heat Stress in Cattle

### 5.1. Effects on Productivity and Reproductive Performance

Physiological mechanisms activated to maintain thermal homeostasis during heat stress inevitably divert energy and nutrients away from productive processes. Reduced dry matter intake, together with the increased energetic cost of thermoregulation, decreases the availability of nutrients for milk synthesis, muscle growth, and reproductive function [31,32]. Consequently, dairy cows exposed to prolonged heat stress experience reductions in milk yield, milk fat and protein concentrations, and lactation persistency, whereas beef cattle exhibit lower average daily gain, reduced feed efficiency, and poorer carcass quality [32,33].

The decline in productive performance is closely associated with the increase in body temperature. Even relatively small elevations in core body temperature may impair rumen function, reduce feed intake, alter endocrine regulation, and modify nutrient partitioning, thereby decreasing production efficiency [31,33]. These responses represent adaptive mechanisms that prioritize survival over productivity during periods of excessive thermal load.

Reproductive performance is particularly sensitive to heat stress because elevated body temperature affects multiple stages of the reproductive process. Heat stress compromises follicular development, reduces oocyte competence, impairs fertilization, increases early embryonic mortality, alters luteal function, and decreases pregnancy rates [34]. In bulls, elevated testicular temperature reduces semen quality through impaired spermatogenesis and increased sperm abnormalities [34].

Evidence also indicates that exposure of pregnant Bos indicus cows to elevated Temperature–Humidity Index (THI) values during late gestation delays postpartum ovarian resumption and reduces the expression of reproductive behaviour after calving [35]. Animals exposed to THI values above 76 exhibit delayed ovarian cyclicity and reduced reproductive efficiency, highlighting the importance of minimizing heat load during critical reproductive periods through appropriate management practices, including shade provision, unrestricted access to drinking water, and active cooling systems [35].

Because productive and reproductive losses result primarily from sustained increases in body temperature, early detection of heat stress using thermoregulatory indicators provides an opportunity to implement timely interventions before irreversible physiological alterations occur.

### 5.2. Effects on Health and Animal Welfare

Heat stress induces profound physiological changes that extend beyond thermoregulation and directly influence animal health. Persistent activation of the hypothalamic–pituitary–adrenal axis and alterations in energy metabolism suppress immune function, reducing leukocyte activity and increasing susceptibility to infectious diseases, particularly mastitis, respiratory disorders, and gastrointestinal diseases [6,13]. In addition, reduced feed intake and altered rumen fermentation increase the risk of metabolic disorders, including ruminal acidosis, dehydration, and electrolyte imbalance, particularly during prolonged periods of elevated environmental temperature.

At the cellular level, oxidative stress and increased production of reactive oxygen species contribute to tissue damage, impaired mitochondrial function, and inflammatory responses [13]. These mechanisms reduce the animal’s capacity to cope with subsequent thermal challenges and may prolong recovery after heat stress episodes.

Animal welfare is compromised when cattle are unable to maintain thermal comfort despite activation of physiological and behavioural adaptations. Heat-stressed cattle spend more time standing to increase convective heat loss, reduce lying time, decrease feed intake and rumination, increase water consumption, and exhibit elevated respiratory effort and panting [6]. Although these behavioural responses facilitate heat dissipation, their persistence indicates that animals remain outside their thermoneutral zone and are experiencing thermal discomfort.

From a practical perspective, monitoring physiological indicators such as rectal temperature, respiratory rate, and surface temperature, together with environmental indices including THI and black globe temperature, enables earlier identification of animals at risk of heat stress. This allows producers to implement targeted mitigation strategies before significant reductions in production, reproductive performance, or animal welfare occur, thereby improving both biological efficiency and economic sustainability in cattle production systems.

## 6. Conclusions

Heat stress remains one of the most important environmental challenges affecting cattle production systems, particularly in tropical and subtropical regions. Cattle respond to elevated environmental temperatures through a coordinated set of physiological, metabolic, endocrine, and thermoregulatory mechanisms aimed at maintaining homeothermy. However, prolonged activation of these responses may negatively affect animal welfare, health, productivity, and reproductive performance.

The field observations conducted in *Bos indicus* cows under tropical conditions in Tabasco, Mexico demonstrated a clear relationship between environmental heat load and physiological responses. Black globe temperatures measured under direct solar radiation were significantly higher than those recorded under shaded conditions, particularly during the hottest hours of the day. Likewise, rectal temperature increased significantly during periods of maximum environmental heat load, indicating that body temperature closely reflects changes in the thermal environment.

These findings support the use of environmental and physiological indicators, particularly black globe temperature and rectal temperature, as practical tools for monitoring heat stress under field conditions. The results also highlight the importance of shade and microclimatic conditions in reducing thermal load and improving the capacity of cattle to maintain thermal balance.

An integrated monitoring approach combining environmental measurements, physiological indicators, and heat stress indices such as the THI can facilitate the early detection of thermal stress and support timely management interventions. The implementation of strategies such as shade provision, adequate water availability, ventilation, and cooling systems is essential to mitigate the adverse effects of heat stress and improve animal welfare, reproductive efficiency, and production sustainability in a warming climate.

## Figures and Tables

**Table 1:** Technical specifications of the instruments used for environmental and physiological temperature measurements.

Instrument	Manufacturer	Variable Measured	Measurement Range	Resolution/Sensitivity	Accuracy
Veterinary rectal thermometer	Sinteg^®^	Rectal (core) body temperature	32–43 °C	0.1 °C	±0.1 °C *
Infrared thermometer Fluke 64 MAX	Fluke Corporation, Everett, WA, USA	Body surface temperature	−30 to 600 °C	0.1 °C; optical resolution 20:1	±1.0 °C or ±1% of reading (whichever is greater)
Dry-bulb mercury thermometer	Laboratory glass thermometer	Ambient air temperature	−10 to 110 °C	1 °C graduation	±1 °C **
Black globe thermometer	Globe thermometer (150-mm matte black copper sphere with centrally positioned mercury thermometer)	Environmental heat load (combined effect of air temperature, solar radiation and wind)	−10 to 110 °C ***	1 °C graduation	±1 °C **

* According to the manufacturer’s specifications. ** Based on the specifications of the laboratory mercury thermometer used (1 °C graduation). Commercial laboratory mercury thermometers of this type have an operating range of −10 to 110 °C and are routinely used for environmental temperature measurements. *** The globe thermometer consisted of a matte black copper sphere containing a centrally positioned mercury thermometer, allowing the integration of the effects of air temperature, solar radiation, and air movement to estimate environmental thermal load.

**Table 2:** Black globe temperature (°C) recorded under different environmental conditions during the period of maximum thermal load (10:30–14:00 h) in Bos indicus cows raised under tropical conditions in Tabasco, Mexico.

Environmental Condition	Temperature (Mean ± SD, °C)	Statistical Group *
Black globe under direct solar radiation (TBGS)	42.12 ± 3.36	a
Black globe under tree shade (TBGT)	33.62 ± 1.51	b
Black globe under roofed shade (TBGR)	33.25 ± 2.05	b

* Values are expressed as mean ± standard deviation. Means with different lowercase letters differ significantly (*p* < 0.05) according to repeated-measures ANOVA followed by the corresponding multiple-comparison test. Significant differences were detected only during the period of highest environmental heat load (10:30–14:00 h). No significant differences (*p* > 0.05) were observed among environmental conditions during the remaining sampling period (14:30–09:00 h of the following day).

**Table 3:** Environmental temperature (°C) recorded using black globe and dry-bulb thermometers under different environmental conditions in a tropical cattle production system in Tabasco, Mexico.

Environmental Condition	Temperature (Mean ± SD, °C)	Statistical Group *
Black globe thermometer under roofed shade (TBGR)	33.17 ± 1.63	a
Dry-bulb thermometer under tree shade (TDBSh)	30.76 ± 1.09	a
Dry-bulb thermometer under direct solar radiation (TDBS)	30.82 ± 2.77	a

* Values are expressed as mean ± standard deviation. Means sharing the same lowercase letter do not differ significantly (*p* > 0.05) according to repeated-measures ANOVA.

**Table 4:** Comparison of environmental temperature (°C) measured using black globe and dry-bulb thermometers under direct solar radiation during the period of maximum environmental heat load (10:00–14:00 h) in a tropical cattle production system in Tabasco, Mexico.

Measurement Method	Temperature (Mean ± SD, °C)	Statistical Group *
Black globe thermometer (TBGS)	42.12 ± 3.36	a
Dry-bulb thermometer (TDBS)	30.13 ± 3.91	b

* Values are expressed as mean ± standard deviation. Means with different lowercase letters differ significantly (*p* < 0.05) according to repeated-measures ANOVA. Significant differences between measurement methods were observed only during the period of highest environmental heat load (10:00–14:00 h). Outside this interval, no significant differences (*p* > 0.05) were detected.

**Table 5:** Rectal and infrared body temperatures of *Bos indicus* cows measured during different periods of the day under tropical environmental conditions in Tabasco, Mexico.

Sampling Period	Rectal Temperature (Mean ± SD, °C)	Infrared Temperature (Mean ± SD, °C)
Morning (09:00–11:00 h)	38.73 ± 0.73 ^b^	36.20 ± 1.13 ^c^
Peak heat load (12:00–14:00 h)	41.24 ± 2.00 ^a^	40.49 ± 2.58 ^d^

Values are expressed as mean ± standard deviation. Different superscript letters indicate significant differences in rectal temperature between sampling periods (*p* = 0.001). Infrared temperatures were significantly lower than rectal temperatures during the period of maximum environmental heat load (*p* < 0.05).

**Table 6:** General classification of heat stress severity in cattle according to the Temperature–Humidity Index (THI) [28].

THI	Heat Stress Level	Expected Physiological Response
<68	No heat stress	Normal thermoregulation
68–71	Mild	Slight increase in respiratory rate
72–78	Moderate	Increased respiratory rate, reduced feed intake
>78	Severe	Hyperthermia, panting, reduced productivity
>84	Emergency	High risk of heat exhaustion

## Data Availability

The data supporting the findings of this study are available from the corresponding author upon reasonable request. The data are not publicly available because they form part of the original doctoral research of the first author.
